# Prognostic Value of Metastatic Lymph Node Ratio and Identification of Factors Influencing the Lymph Node Yield in Patients Undergoing Curative Colon Cancer Resection

**DOI:** 10.3390/cancers16010218

**Published:** 2024-01-02

**Authors:** Paweł Mroczkowski, Samuel Kim, Ronny Otto, Hans Lippert, Radosław Zajdel, Karolina Zajdel, Anna Merecz-Sadowska

**Affiliations:** 1 Department for General and Colorectal Surgery, Medical University of Lodz, Pl. Hallera 1, 90-647 Lodz, Poland; pawel.mroczkowski@umed.lodz.pl; 2 Institute for Quality Assurance in Operative Medicine Ltd., Otto-von-Guericke-University, Leipziger Str. 44, 39120 Magdeburg, Germany; samuel.kim@gmx.net (S.K.); ronny.otto@med.ovgu.de (R.O.); hans.lippert@med.ovgu.de (H.L.); 3 Department for Surgery, University Hospital Knappschaftskrankenhaus, Ruhr-University, In der Schornau 23-25, 44892 Bochum, Germany; 4 Sanitätsversorgungszentrum Torgelow, Bundeswehr Neumühler Str. 10b, 17358 Torgelow, Germany; 5 Department for General, Visceral and Vascular Surgery, Otto-von-Guericke-University, Leipziger Str. 44, 39120 Magdeburg, Germany; 6 Department of Economic and Medical Informatics, University of Lodz, 90-214 Lodz, Poland; radoslaw.zajdel@uni.lodz.pl; 7 Department of Medical Informatics and Statistics, Medical University of Lodz, 90-645 Lodz, Poland; karolina.smigiel@umed.lodz.pl; 8 Department of Allergology and Respiratory Rehabilitation, Medical University of Lodz, 90-725 Lodz, Poland

**Keywords:** colon cancer, lymph nodes, lymph node yield, lymph node ratio, five-year overall survival

## Abstract

**Simple Summary:**

In patients with colon cancer, the number of lymph nodes examined during surgery can have a significant impact on their long-term survival. We conducted a study with over 7000 patients and found that those who had at least 12 lymph nodes evaluated had better survival rates. A younger age, specific cancer stages, and a right-sided tumor location were associated with a higher number of lymph nodes examined. Additionally, we discovered that the ratio of metastatic to examined nodes (LNR) was a valuable predictor of survival and provided more precise information than the conventional pN classification system. This research emphasizes the importance of a thorough lymph node evaluation in colon cancer patients for accurate prognosis and treatment decisions.

**Abstract:**

Due to the impact of nodal metastasis on colon cancer prognosis, adequate regional lymph node resection and accurate pathological evaluation are required. The ratio of metastatic to examined nodes may bring an additional prognostic value to the actual staging system. This study analyzes the identification of factors influencing a high lymph node yield and its impact on survival. The lymph node ratio was determined in patients with fewer than 12 or at least 12 evaluated nodes. The study included patients after radical colon cancer resection in UICC stages II and III. For the lymph node ratio (LNR) analysis, node-positive patients were divided into four categories: i.e., LNR 1 (<0.05), LNR 2 (≥0.05; <0.2), LNR 3 (≥0.2; <0.4), and LNR 4 (≥0.4), and classified into two groups: i.e., those with <12 and ≥12 evaluated nodes. The study was conducted on 7012 patients who met the set criteria and were included in the data analysis. The mean number of examined lymph nodes was 22.08 (SD 10.64, median 20). Among the study subjects, 94.5% had 12 or more nodes evaluated. These patients were more likely to be younger, women, with a lower ASA classification, pT3 and pN2 categories. Also, they had no risk factors and frequently had a right-sided tumor. In the multivariate analysis, a younger age, ASA classification of II and III, high pT and pN categories, absence of risk factors, and right-sided location remained independent predictors for a lymph node yield ≥12. The univariate survival analysis of the entire cohort demonstrated a better five-year overall survival (OS) in patients with at least 12 lymph nodes examined (68% vs. 63%, *p* = 0.027). The LNR groups showed a significant association with OS, reaching from 75.5% for LNR 1 to 33.1% for LNR 4 (*p* < 0.001) in the ≥12 cohort, and from 74.8% for LNR2 to 49.3% for LNR4 (*p* = 0.007) in the <12 cohort. This influence remained significant and independent in multivariate analyses. The hazard ratios ranged from 1.016 to 2.698 for patients with less than 12 nodes, and from 1.248 to 3.615 for those with at least 12 nodes. The LNR allowed for a more precise estimation of the OS compared with the pN classification system. The metastatic lymph node ratio is an independent predictor for survival and should be included in current staging and therapeutic decision-making processes.

## 1. Introduction

Colorectal cancer (CRC) is a complex and multifactorial disease with a significant global impact. It ranks as the third most frequently diagnosed cancer and the second leading cause of cancer-related mortality worldwide. Based on the site of onset, rectal cancer comprises 49.66% of cases, whereas colon cancer accounts for 49.09%. When considering both sites together, they collectively represent 1.25% of all cases. 

The exact causes of CRC remain uncertain, although they may be associated with various factors such as genetic and dietary elements, as well as noncancerous health conditions. The risk of CRC rises with advancing age. Incidence and mortality rates for CRC are relatively low up to the age of 45; however, later they significantly increase. The highest incidence is observed in the age group over 80 years. Nonetheless, a noteworthy number of cases may still be observed among adolescents. 

The epithelial cells of the mucosa in the colon and rectum can go through various stages of development, including hyperplasia, atypical hyperplasia, and adenomas. These adenomas have the potential to progress into carcinomas. In the early stages of CRC, the disease is usually limited to the mucosa and submucosa of the intestinal wall, and lymphatic metastasis is rare at this point. However, when the tumor penetrates the submucosal layer, lymphatic metastasis can occur. CRC usually metastasizes to the liver, lungs, lymph nodes of the abdominal cavity, and the peritoneum [1,2,3,4]. 

CRC is a complex, multi-step disease whose development depends on the accumulation of genetic and epigenetic alterations. These include the loss of tumor suppressor function (including APC and p53) and activation of proto-oncogenes (including KRAS and BRAF). Such molecular derangements ultimately lead to dysregulated cell proliferation, inhibited apoptosis, and the activation of growth-promoting signaling pathways [5,6,7].

Circulating tumor DNA (ctDNA) refers to fragmented DNA from tumor cells that is released into the bloodstream. In metastatic CRC, ctDNA enables noninvasive molecular profiling to identify actionable biomarkers and guide targeted therapy decisions. Specifically, ctDNA analysis can effectively determine the mutation status, microsatellite instability, and tumor mutational burden. However, tissue biopsy remains the gold standard, with a higher sensitivity for detecting certain genomic alterations. But for CRC, ctDNA has high detection rates nearing 100% in metastatic disease. Ongoing studies continue to evaluate concordance between ctDNA and tissue sequencing across various genomic biomarkers. Overall, ctDNA is becoming an invaluable tool for genotyping and tracking tumor dynamics in CRC [8,9,10].

Early stages of CRC often give no symptoms. As the disease progresses, patients typically experience symptoms such as hematochezia, intestinal obstruction, abdominal mass, and various systemic symptoms. The five-year overall survival (OS) rate varies depending on the disease stage, with a rate of 90% at stage I, 70–80% at stage II, and 40–65% at stage III. The risk of progression also correlates with the stage of the primary tumor; namely, it is 30% for stage II and 50% for stage III. Additionally, the risk is higher in the first two years following radical surgery [11,12].

This work focuses on colon cancer patients. The management of colon cancer patients is primarily determined by the stage of the disease at diagnosis, underscoring the need for a thorough approach to diagnosing, assessing, and treating the condition. Adequate lymphadenectomy, recently described in the concept of a complete mesocolic excision (CME), remains a crucial element of surgical treatment in nonmetastatic colon cancer [13]. The removal and analysis of lymph nodes play both a therapeutic and prognostic role. The involvement of the lymph nodes determines the stage of the disease, its prognosis and potential indication for adjuvant strategies [11,14].

The current standard of care for stage III colon cancer is immediate resection followed by adjuvant chemotherapy. Adjuvant chemotherapy has been shown to reduce the risk of recurrence and improve the OS in this patient population. Over the past decades, several landmark trials have established the efficacy of various chemotherapeutic regimens in the adjuvant setting [15,16,17]. Initially, studies demonstrated the efficacy of adjuvant fluorouracil (5-FU) and folinic acid in colon cancer. IMPACT investigators demonstrated the benefits of using 5-FU and folinic acid, increasing the OS from 78% to 83% [18]. The addition of oxaliplatin to 5-FU/folinic acid (FOLFOX regimen) was then validated as more effective than 5-FU regimens alone, becoming the new standard of care. The addition of oxaliplatin was first suggested with the MOSAIC trial, showing a significantly improved six-year OS rate of 78.5% compared to 72.9% with 5-FU alone [19]. More recently, oral fluoropyrimidines like capecitabine combined with oxaliplatin (CAPOX) have shown similar improvements in patient outcomes. The XELOXA trial found that combination therapy with capecitabine and oxaliplatin was superior to 5-FU alone, with a 5-year OS rate of 73% compared to 67% [20]. Thus, an oxaliplatin-based doublet therapy with 5-FU/folinic acid or capecitabine is now the backbone of adjuvant treatment for resected stage III colon cancer [15,16,17].

Accurate lymph node resection, analysis, and examination (LNE) are crucial in predicting the future outcomes of patients who underwent radical surgery for colon cancer [21]. According to the guidelines issued by the American Joint Committee on Cancer (AJCC), it is recommended to assess a minimum of 12 lymph nodes in order to meet the threshold requirement [22]. In the case of lymph node involvement (stage III colon cancer), there is a risk of misclassification into stages I or II, if the number of LNEs is insufficient. Such misclassification may result in patients not receiving the appropriate adjuvant therapy. Therefore, in recent reports, it has been indicated that an increased number of LNEs correlates with improved prognosis [23,24,25,26].

In the seventh edition of classification of malignant tumors (TNM), the AJCC introduced a subdivision of the N parameter for colon cancer, which includes N1a (only 1 metastatic node), N1b (2–3 positive nodes), N2a (4–6 positive lymph nodes), and N2b (≥7 positive lymph nodes). However, the number of LNEs is still not part of the TNM staging system [27,28]. Therefore, there have been suggestions to use the lymph node ratio (LNR) as an improvement in the staging of CRC. The LNR is determined by calculating the ratio of metastatic lymph nodes to the total number of resected lymph nodes. It is believed that the LNR has the potential to serve as a more accurate prognostic factor for CRC compared to the conventional N assessment within the current TNM staging system [29,30].

The present study investigated large real-life population-based cohorts undergoing colectomies for cancer to evaluate factors influencing the achievement of the 12 lymph node limit as well as the prognostic impact of the LNR, in comparison with the actual N-classification within the TNM staging system.

## 2. Materials and Methods

### 2.1. Patient Population

The study analyzed the complete data of 7012 patients treated for colon cancer in 122 hospitals that participated in an observational study entitled “Quality Assurance in Colorectal Cancer,” managed by the An-Institute at the Otto von Guericke University Magdeburg, Germany in the years 2008–2012. Patients with UICC (Union Internationale Contre le Cancer, Geneva, Switzerland) stage II and III colon adenocarcinoma who underwent radical tumor resection were included. The colon was defined as the segment of the bowel between >16 cm from the anocutaneous line and ileocolic valve. Curative resection was defined as the complete resection of a macroscopic tumor with negative pathological margins, lymphadenectomy, and no evidence of metastases. Patients with rectal cancers, multiple colon cancers, and second primary tumors were excluded from the study.

Since it was an observational study, no ethical approval was required, as confirmed by the local ethics committee of the Otto von Guericke University Magdeburg. Written informed consent was obtained from each patient.

### 2.2. Data Collection

The hospitals were required to deliver data on every patient treated for colon cancer. The total number of reported patients was cross-checked with the hospital’s financial report for insurance companies to avoid a selection bias. The enrolment questionnaire consisted of 68 questions related to personal data, risk factors, reasons for hospitalization, diagnosis prior to surgery, surgical procedure, surgery-related complications, results of pathology tests, and discharge (total: 334 items). Risk factors were defined based on the assessment prior to the surgical treatment and categorized as follows: none, cardiac, respiratory, renal, hepatogenic, nicotine abuse, alcohol abuse, diabetes mellitus, varicosis, and others. Each patient’s body mass index (BMI) and American Society of Anesthesiologists (ASA) score were also recorded. The surgical procedures were classified by a surgeon and divided into categories including right hemicolectomy, extended right hemicolectomy, left hemicolectomy, extended left hemicolectomy, and sigmoid resection. The intraoperative course was described by the duration of the surgery, presence and technique of anastomosis, and intraoperative complications (bladder injury, bleeding necessitating > 2 red blood cell concentrates, ureter lesion, iatrogenic tumor perforation, spleen injury, intestinal injury, internal genital injury, problem regarding the capnoperitoneum, and anastomosis complication). The postoperative complications included general and special ones. The general postoperative complications were lung embolism, pulmonary problems (pleural effusion and atelectasis), pneumonia, urinary tract infection, fever (>38 °C, >2 days), cardiac problems, multiple organ failure, thrombosis, and renal problems. The postoperative special complications were bleeding (necessitating surgery), wound abscess, sepsis, anastomosis insufficiency, aseptic wound healing dysfunction, wound infection, intra-abdominal⁄retrorectal abscess, mechanical ileus (necessitating surgery), fecal fistula, peritonitis, atony lasting longer than three days, peristalsis dysfunction (not necessitating surgery), wound dehiscence, and colostomy complication. The number of resected regional lymph nodes and UICC classification were recorded based on the pathological report. Survival data were collected by review of medical records and comparison with available registers.

### 2.3. Statistical Analysis

In this analysis, constant variables were used with appropriate measurements and given as the mean with standard deviation, minimum and maximum or as the median, minimum and maximum. Categorical variables were displayed as absolute or relative frequencies. The chi-square test was used to proof the independency of categorical variables. For small sample numbers (<5), cross-tabulation or Fisher’s exact test were used. For estimations of systematic differences between the groups, a test of normal distribution was performed (the Shapiro–Wilk test). In the first step, factors influencing lymph node yield (LNY) were analyzed univariately. The independence of the significant factors was verified in a multivariate regression and displayed as an odds ratio (OR) with a 95% confidence interval. For survival analysis, the patient population was divided in two groups: i.e., those with <12 and ≥12 examined lymph nodes, in order to exclude potential bias of a low LNY. In univariate survival analysis, the previously identified significant factors influencing LNY were tested according to the Kaplan–Meier method, using the log-rank test. The nodal positive subgroup was divided into four categories: LNR 1 (<0.05), LNR 2 (≥0.05; <0.2), LNR 3 (≥0.2; <0.4), and LNR 4 (≥0.4), as initially proposed by Berger et al. [21]. For multivariate survival analysis, the method of Cox regression was used. The specified hazard ratios (HR) were also given with 95% confidence intervals. All statistical comparisons were performed at the significance level of 5%. Statistical analysis was performed using IBM^®^ SPSS^®^ Statistics, Version 21.0.0, SPSS Inc. (New York, NY, USA).

## 3. Results

The main data analysis included 7012 patients with UICC stage II and III colon cancer who met the set criteria. The mean number of examined lymph nodes was 22.08 (SD 10.64, median 20). In the study group, 94.5% had 12 or more nodes evaluated. In patients with an LNE < 12, an average of 8.99 (95% CI: 8.78–9.21) nodes were analyzed by the pathologist and 1.14 (95% CI: 0.96–1.32) identified as positive, while in those with an LNE ≥ 12, an average of 22.84 (95% CI: 22.58–23.09) lymph nodes were analyzed and 1.93 (95% CI: 1.83–2.03) were found to be metastatic. Patients with 12 or more nodes were more likely to be younger, women, with a lower ASA classification, pT3 and pN2 categories, and had no risk factors. Additionally, an association with right-sided tumor location was observed as well (Table 1).

In the multivariate analysis (Table 2), an age < 50, ASA classification of II and III, pT2, pT3, pT4, and pN2 categories, and absence of risk factors remained independent predictors for a LNY ≥ 12, as well as the right-sided location.

**Figure 1:**
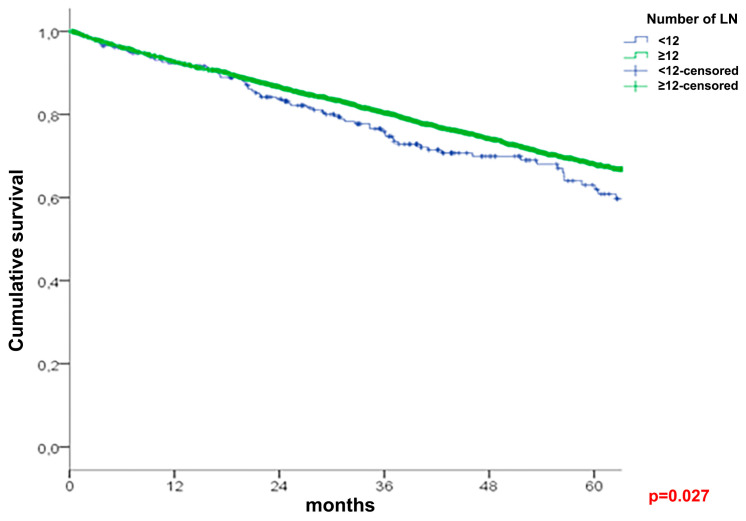
Overall survival in patients with <12 and ≥12 evaluated nodes.

**Figure 2:**
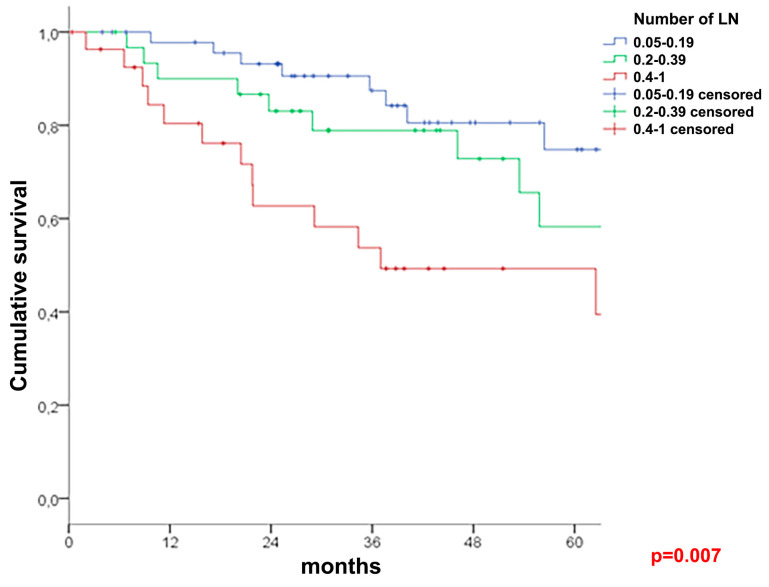
Overall survival in patients with <12 evaluated nodes.

**Figure 3:**
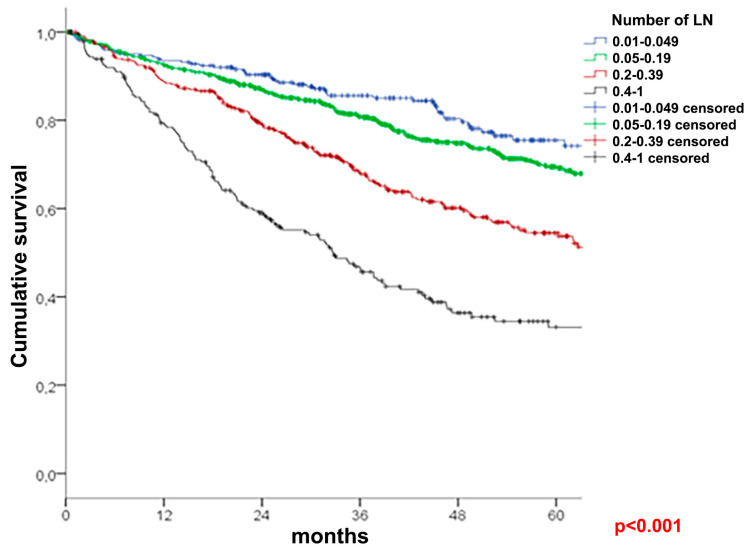
Overall survival in patients with ≥ 12 evaluated nodes.

A univariate survival analysis of the entire cohort (Table 3) demonstrated a better five-year OS in patients with at least 12 lymph nodes examined (68% vs. 63%, *p* = 0.027, Figure 1). The LNR groups (N = 183 for <12 LNY cohort: N = 85 for LNR 2, N = 56 for LNR 3, and N = 42 for LNR 4; N = 3081 for ≥12 LNY cohort: N = 462 for LNR1, N = 1599 for LNR 2, N = 622 for LNR 3, and N = 398 for LNR 4) showed a significant association with the OS reaching from 75.5% for LNR 1 to 33.1% for LNR 4 (*p* < 0.001) in the ≥12 cohort, and from 74.8% for LNR 2 to 49.3% for LNR 4 (*p* = 0.007) in the <12 cohort (Figure 2 and Figure 3). This influence remained significant and independent from multivariate analyses.

The hazard ratios ranged from 1.016 to 2.698 for patients with less than 12 lymph nodes and from 1.248 to 3.615 for those with at least 12 lymph nodes. The LNR allowed a more precise estimation of the OS compared with the pN classification system for LNR 4 in the group with <12 lymph nodes and LNR 3 and LNR 4 in the group with ≥12 lymph nodes (Table 4).

## 4. Discussion

The LNR and pN classification system were independent prognostic factors in both cohorts (<12 and ≥12 nodes). Comparing the HR and OS, the LNR in patients with ≥12 lymph nodes appears to give a more accurate prognosis than the pN categories: HR (LNR 4) = 3.615 vs. HR (pN2) = 1.832, while OS (LNR 4) = 33.1% vs. OS (pN2) = 49.0%. LNR 4 better predicts the OS than the pN2 category when at least 12 nodes are evaluated. These findings are complementary to previous references showing the LNR providing additional staging information to the current staging system. Berger et al., whose cutoffs were adopted in this study, showed the LNR to be a significant prognostic variable in stage II and III if at least 10 nodes were evaluated [31]. However, this initial cohort was significantly smaller (n = 3411) than in the present analysis, and the included patients were part of a randomized controlled trial, not a cohort from a real-life treatment. When analyzing 922 single-center colon cancer patients in stage III, Parnaby et al. demonstrated the superiority of LNR cutoffs of 18%, 42%, and 70% compared to the pN classification system using the Akaike information criterion [32]. In a Danish nationwide study including 8901 patients operated on for nonmetastatic colon cancer (incl. 1263 stage I cases), Lykke et al. showed an association of a high LNY with improved survival, as well as a prognostic advantage of the LNR compared to the pN classification system [33]. Chen et al. analyzed 36,712 colon cancer patients from the administrative National Cancer Institute Surveillance, Epidemiology, and End Results (SEER) database for the years 1992–2004. Similar to our findings, the authors noticed the best prognostic value of LNRs after an LNE ≥ 12, whereas the multivariate analysis suggested the ratio to be a better prognostic factor than the pN classification system [34]. Silva et al. demonstrated that the LNR is a strong predictor for tumor recurrence in stage III colon cancer [35]. Moreover, according to Jang et al., the LNR holds the potential to serve as an autonomous prognostic element for patients with stage IV colon cancer who undergo resection [36]. Additional evidence comes from Mirzaei et al. [37], who demonstrated that LNRs had significant prognostic value for both overall and disease-free survival in stage III colon cancer. Amri et al. [38] reported significant associations of LNRs with cancer-related mortality and recurrence in a cohort of over 1000 patients. Occhionorelli et al. [39] found LNRs to predict the 5-year overall and disease-free survival in emergency colon cancer surgery. In the study by Elbaiomy et al. [40], a high LNR was significantly associated with poorer progression-free and overall survival. Other authors, such as Jakob et al., Schiffman et al., or Mohan et al., could not find any additional prognostic value of LNRs [30,41,42]. However, due to a low number of cases (144–402 patients) and the subdivision of LNRs with only one cutoff, these results have a limited impact.

In our patient cohort with UICC II–III colon cancer, on average, 22.08 (SD 10.645, median 20) lymph nodes were examined. The correlation between LNE and a better OS has already been shown by multiple studies [43]. Foo et al. suggest that the LNY shows a substantial correlation with survival outcomes. A lymph node yield of 20 or more was linked to improved survival. Conversely, a lymph node yield of less than 12 did not demonstrate inferior survival outcomes when compared to those with node yields between 12 and 19 [44]. Lykke et al. propose that in UICC stage I–III colon cancer, a LNY exceeding the recommended 12 lymph nodes was linked to enhanced survival [45]. Our results show a significantly increased survival in patients with ≥12 nodes. Yet, the exact reason for this phenomenon is still uncertain. The evaluation of at least 12 nodes, recommended by numerous guidelines, is supposed to ensure accurate staging and prevent possible understaging and undertreatment. Lykke et al. observed stage migration in UICC III patients with more than 12 nodes evaluated [33]. Our results support this finding: node positive patients were more likely to be ranked into the pN2 category when at least 12 nodes were examined, while the pN1 category was more represented in patients with <12 nodes (*p* < 0.001). Also, the OS in both pN categories was higher in ≥12 nodes. Other authors reject this theory [46,47,48,49,50]. Budde et al. observed no improvement in staging despite the increasing number of examined nodes from 2004 to 2010 [51]. Another theory is that an increased number of nodes is found in patients with a better immunologic response to the tumor, which leads to increased survival [46,48,51,52,53]. Another additional explanation is that the LNE serves as proxy for quality of surgery and pathology [6,54].

According to our analysis, a younger age was accompanied by OR 4.7 for a LNY ≥ 12. Other studies confirmed this finding [55,56,57,58,59,60]. Chou et al. demonstrated a 9% reduction in LNY for each ten-year interval [59]. Lykke et al. and Nathan et al. observed a decreased OR in older patients (OR 1 to 0.452 and OR 1 to 0.720) [57,58]. This phenomenon results from an insufficient immune response in older patients [57,61]. Another explanation might be the risk reduction of the surgery at the cost of the LNY, prioritizing the minimization of anesthesia thanks to the shorter duration of the surgical procedure in older patients who are often dealing with comorbidities [59]. ASA classifications of II and III were associated with an adequate LNY, while Moro-Valdezate and Nash et al. did not find any statistical correlation [62,63]. In our multivariate analyses, female patients had no statistical benefit for a high LNY. Some authors showed a correlation with the female sex [57,58,61,64], whereas others did not obtain such results [46,63,65,66,67]. In the present study, no difference was found in the laparotomic and laparoscopic approaches, which confirmed the results of Beccera and Lykke et al. [56,57]

According to our study, a right-sided tumor location was beneficial for a LNY ≥ 12 (OR 2.3), which was congruent with other studies [56,57,63,68,69,70,71,72]. The left-sided tumor location was described by Becerra et al. as a risk factor for a yield < 12 (OR 1.158), and with an OR of 5.7–6.7 and even a high-risk factor by Choi et al. [56,64]. Some authors believe it is related to a variable lymphatic anatomy: lymph nodes are more likely to be found along the right-sided ileocolic artery than along the left-sided vessels [57,59,63]. Genetic–immunological causes are considered as well. Microsatellite instability was mainly associated with right-sided tumors. These types of tumors are more amenable to the immune system, resulting in higher yields [73,74].

In multivariate regression, a high pT category was associated with an LNE ≥ 12: especially pT3 tumors were found to have an OR of 4.6. Other studies report similar conclusions. Lykke et al. as well as Nathan et al. showed an increasing OR with increasing pT categories for an adequate yield [57,58]. In a Korean study, a low pT category was a risk factor for inadequate yield [64]. A proposed explanation is that tumor necrosis, which is more frequently found in a high pT category, leads to a higher antigen presentation for the immune system, resulting in an increased lymph node yield [57].

This study has several limitations. A multicentric study is based on voluntary participation of hospitals and family physicians, without the discipline and resources of randomized controlled trials. Also, due to the high rate of adequate LNEs, the cases < 12 nodes are low, which must be taken into account when interpreting this part of the results. Our data do not include information about oncological treatments administered after surgery–adjuvant chemotherapy, further resections, or palliative chemotherapy for metastatic disease.

## 5. Conclusions

The LNR allows for a better estimation of the overall survival compared to the pN status and shows remarkable differences in prognosis within nodal-positive patients. It is unclear why the LNR still remains outside of the UICC stage classification for colon cancer and is not included in the decision-making process concerning adjuvant therapies.

## Figures and Tables

**Table 1:** Univariate analysis of lymph node harvest <12 and ≥12.

	<12 N (%)	≥12 N (%)	*p*-Value
**Age**			
<50	3 (0.8)	278 (4.2)	0.018
50–60	44 (11.5)	842 (12.7)	
61–70	101 (26.1)	1643 (24.8)	
71–80	140 (36.3)	2425 (36.6)	
81–90	91 (23.5)	1332 (20.1)	
>90	7 (1.8)	106 (1.6)	
**Sex**			
Male	225 (58.4)	3472 (52.4)	0.027
Female	161 (41.6)	3154 (47.6)	
**ASA Classification**			
I	21 (5.4)	417 (6.3)	<0.001
II	157 (40.6)	3114 (47.0)	
III	181 (47.0)	2889 (43.6)	
IV	27 (7.0)	206 (3.1)	
**pT Category**			
pT1	20 (5.1)	80 (1.2)	<0.001
pT2	29 (7.5)	278 (4.2)	
pT3	264 (68.5)	5036 (76.0)	
pT4	73 (18.9)	1232 (18.6)	
**pN Category**			
pN0	203 (52.5)	3545 (53.5)	<0.001
pN1	147 (38.1)	1955 (29.5)	
pN2	36 (9.4)	1126 (17.0)	
**Risk Factors**			
At least one	322 (83.4)	5115 (77.2)	0.004
None	64 (16.6)	1511 (22.8)	
**Tumor Location**			
Caecum	51 (13.1)	1199 (18.1)	0.011
Colon ascendens	36 (9.4)	1411 (21.3)	<0.001
Colon descendens	36 (9.4)	378 (5.7)	0.005
Colon sigmoideum	187 (48.3)	2319 (35.0)	<0.001
Flexura dextra	16 (4.2)	484 (7.3)	0.019
Flexura sinistra	21 (5.5)	305 (4.6)	0.449
Colon transversum	39 (10.1)	530 (8.0)	0.075
**UICC**			
II	203 (52.5)	3545 (53.5)	0.682
III	183 (47.5)	3081 (46.5)	
**Grading**			
G1	8 (2.1)	146 (2.2)	0.490
G2	288 (74.7)	4777 (72.1)	
G3	88 (22.7)	1690 (25.5)	
G4	2 (0.5)	13 (0.2)	
**Access**			
Laparotomy	331 (85.6)	5599 (84.5)	0.276
Laparoscopy	25 (6.5)	411 (6.2)	
Laparoscopic-assisted	19 (5.0)	477 (7.2)	
conversion	11 (2.9)	139 (2.1)	
**Intraoperative Complications**			
At least one	11 (2.9)	166 (2.5)	0.661
None	375 (97.1)	6460 (97.5)	

**Table 2:** Multivariate analysis of lymph node harvest ≥12.

	Odds Ratio	95% CI	*p*-Value
**Age**			
≥50	Referent		
<50	4.687	1.474–14.900	0.009
**ASA Classification**			
I	1.982	0.971–4.045	0.060
II	2.335	1.457–3.744	<0.001
III	1.994	1.261–3.152	0.003
IV	Referent		
**pT Category**			
pT1	Referent		
pT2	2.177	1.134–4.178	0.019
pT3	4.682	2.684–8.166	<0.001
pT4	3.490	1.934–6.297	<0.001
**pN Category**			
pN0	Referent		
pN1	0.960	0.743–1.241	0.757
pN2	1.788	1.228–2.604	0.002
**Risk Factor**			
At least one	Referent		
None	1.466	1.030–2.087	0.034
**Tumor Location**			
Left side	Referent		
Right side	2.309	1.805–2.955	<0.001
Caecum transversum	1.042	0.721–1.508	0.825

**Table 3:** Univariate survival analysis for lymph node yield <12 and ≥12. Numbers in percentages.

	<12		≥12	
	5-Years-OS in %	*p*-Value	5-Years-OS in %	*p*-Value
**LNR**				
LNR 1	-	0.007	75.5	<0.001
LNR 2	74.8		69.5	
LNR 3	58.3		54.5	
LNR 4	49.3		33.1	
**Sex**				
Male	60.9	0.762	67.2	0.591
Female	67.1		68.9	
**ASA Classification**				
I	64.8	0.001	81.6	<0.001
II	74.6		75.5	
III	53.7		56.7	
IV	42.8		45.7	
**pT Category**				
pT1	75.8	0.004		
pT2	78.4		92.6	<0.001
pT3	67.0		80.7	
pT4	39.9		71.4	
**pN Category**				
pN1	68.2	0.005	70.7	<0.001
pN2	46.7		49.0	
**Risk Factors**				
At least one	60.6	0.046	63.4	<0.001
None	72.5		83.8	
**Tumor Location**				
Right side	50.3	0.111	64.1	<0.001
Left side	66.0		71.5	
Caecum transversum	73.8		70.8	
**Intraoperative Complications**				
At least one	53.6	0.492	64.4	0.329
None	63.3		68.1	
**Morbidity**				
No	67.6	0.049	71.4	<0.001
Yes	55.3		61.3	

**Table 4:** Cox multivariate models for 5-year OS.

	<12 Nodes		≥12 Nodes	
	Hazard Ratio (95% CI)	*p*-Value	Hazard Ratio (95% CI)	*p*-Value
**LNR**				
LNR 1			Referent	
LNR 2	Referent		1.248 (0.922–1.828)	0.152
LNR 3	1.016 (0.363–2.840)	0.976	1.976 (1.428–2.734)	<0.001
LNR 4	2.698 (1.083–6.718)	0.033	3.615 (2.589–5.047)	<0.001
**Age**		ns	1.046 (1.036–1.057)	<0.001
**ASA Classification**				
I	Referent	0.102	Referent	
II	0.273 (0.47–1.571)	0.146	1.120 (0.687–1.828)	0.649
III	0.672 (0.107–4.236)	0.672	1.720 (1.048–2.824)	0.032
IV	0.731 (0.091–5.872)	0.768	2.527 (1.338–4.773)	0.004
**pT Category**				
pT1	Referent		Referent	
pT2	0.969 (0.164–5.722)	0.972	2.113 (0.639–6.989)	0.220
pT3	0.813 (0.182–3.634)	0.787	3.316 (1.060–10.373)	0.039
pT4	3.578 (0.743–17.223)	0.112	5.997 (1.907–18.861)	0.002
**pN Category**				
pN1	Referent		Referent	
pN2	2.957 (1.362–6.421)	0.006	1.832 (1.540–2.179)	<0.001
**Tumor Location**				
Left		ns	Referent	
Right			1.384 (1.151–1.664)	0.001
Caecum transversum			1.177 (0.830–1.670)	0.361
**Morbidity**		ns	1.324 (1.106–1.585)	0.002

ns = not significant (*p* > 0.05).

## Data Availability

Data are contained within the article.
